# Are one-year changes in adherence to the 24-hour movement guidelines associated with depressive symptoms among youth?

**DOI:** 10.1186/s12889-020-08887-z

**Published:** 2020-05-27

**Authors:** Karen A. Patte, Guy Faulkner, Wei Qian, Markus Duncan, Scott T. Leatherdale

**Affiliations:** 1 Department of Health Sciences, Faculty of Applied Health Sciences, Brock University, Niagara Region, 1812 Sir Isaac Brock Way, St. Catharines, Ontario L2S 3A1 Canada; 2 School of Public Health and Health Systems, University of Waterloo, 200 University Ave, Waterloo, Ontario N2L 3G1 Canada; 3 School of Kinesiology, Faculty of Education, University of British Columbia, Vancouver, British Columbia V6T 1Z4 Canada

**Keywords:** Sleep, Physical activity, Screen time, Mental health, Adolescents, Sedentary behaviour

## Abstract

**Background:**

There remains a need for prospective research examining movement behaviours in the prevention and management of mental illness. This study examined whether changes in adherence to the 24-h Movement Guidelines (moderate-to-vigorous physical activity [MVPA], sleep duration, screen time) were associated with depression symptoms among youth.

**Methods:**

Conditional change models were used to analyze two waves of longitudinal questionnaire data (2016/17, 2017/18) from students in grades 9–12 (*N* = 2292) attending 12 schools in Ontario and British Columbia, Canada, as part of the COMPASS study. One-year change in adherence to the MVPA, screen time, and sleep duration guidelines were modeled as predictors of depressive symptoms, adjusting for covariates and prior year depressive symptoms. Models were stratified by sex.

**Results:**

Continued adherence to sleep guidelines and transitioning from inadequate to sufficient sleep were associated with lower depressive symptoms than continued nonadherence, and continued adherence was associated with lower depression than transitioning from sufficient to short sleep. For screen time, transitioning from exceeding guidelines to guideline adherence was associated with lower depressive symptoms than continued nonadherence. MVPA guideline adherence was not associated with depression scores, when controlling for sleep and screen time guideline adherence change and covariates. When combined, meeting additional guidelines than the year prior was associated with lower depressive symptoms among females only.

**Conclusions:**

Adherence to the sleep guidelines emerged as the most consistent predictor of depression symptoms. Promoting adherence to the Movement Guidelines, particularly sleep, should be considered priorities for youth mental health at a population level.

## Background

Depressive disorders are among the most commonly occurring mental illnesses that typically develop in adolescence [1]. Approximately one in ten adolescents will experience a major depressive episode (MDE) [2–4]. Another 18–40% of youth report subclinical depressive symptoms, which increase in frequency and severity over adolescence [5, 6], and are strong predictors of future clinical depressive episodes [7]. With each MDE and increased episode duration, reoccurrence becomes more likely and likelihood of remission declines [8, 9]. About three-quarters of youth experience another MDE within five years [10], and the elevated risk persists into adulthood [1, 11, 12]. Without effective intervention, depression has substantial health, social, and economic consequences [4, 12–14], as the leading global cause of years lived with disability, and second leading cause of disability-adjusted life years [15]. As chronic, reoccurring, and disabling conditions, prevention and early intervention of depressive disorders are critical.

Physical activity (PA) is proposed as a low-cost and undervalued strategy for the prevention and management of depression at the population level [16]. Substantial research has established PA as a first-line intervention for mild-to-moderate depression among adults [17, 18], and a lesser, but growing, evidence base supports the benefits of PA for youth with major depressive disorder (MDD) [19]. In a recent umbrella systematic review [19]*,* increased PA was associated with decreased depression symptoms among children and youth in 12 of the 17 included reviews; however, many reviews were primarily cross-sectional studies, and larger effects tended to occur in clinical samples. Similarly, an updated review of reviews on PA and mental health in children and adolescents reported moderate effect sizes in intervention studies and small or null associations in observational studies [20]. Biddle et al. concluded there was currently insufficient evidence to support temporal sequencing [20]; hence, there is a continued need for prospective evidence of PA in the prevention of depressive disorders at a population level.

Distinct from physical inactivity, sedentary behaviour is defined as any waking behavior characterized by an energy expenditure ≤1.5 metabolic equivalents, while in a sitting, reclining, or lying posture [21]. Screen-based activities, such as computer, television, video game, and mobile device use, represent common forms of sedentary behaviours in youth [22, 23]. Much debate has centered around the potential impact of screen use on youth mental health. Cross-sectional associations between screen time (ST) and depressive symptoms are well established [24–26]; however, longitudinal evidence has been more mixed [27, 28]. Several studies have suggested depressive symptoms predict increased sedentary behaviour, or social media engagement, rather than the reverse [29–32]. Furthermore, a 2019 review of reviews identified most evidence as dated and not reflective of modern screen use, particularly mobile devices and computer/video games [33]. Other key critiques of the extant literature include the reliance on cross-sectional data [28, 34–36], the focus on physical health [35], and inadequate consideration of gender differences [28].

Another leading concern regarding youth screen use is the potential to delay and interrupt sleep. While improving, sleep is often regarded as an underrecognized and undervalued public health issue [37, 38]. Depression research has predominantly focused on sleep disturbance in clinical samples. At a population level, some evidence suggests sleep-related developmental changes partially mediate increases in depressive symptoms across adolescence [39, 40]. Indeed, the rise in depressive symptoms and depression onset over adolescence parallels declining sleep duration trajectories [39]. However, while cross-sectional associations between short sleep and depressive symptoms are well established [39, 41–44], a paucity of longitudinal population-level research exists and available prospective evidence is inconsistent. Some results suggest a bi-directional relationship or reverse causality [29, 31, 45], and others signify sex and gender differences, often with inverse associations between sleep duration and depressive symptoms in females only, and weaker or null effects in males [46, 47].

While prior research has often focused on one or two movement behaviours, researchers now recognize the need to consider all movement behaviours as codependent and distinct determinants of health [48]. Reflecting this shift, the *Canadian 24-h Movement Guidelines for Youth* provide integrated and evidence-based recommendations on PA, sedentary behaviours, and sleep across a day [21]. Based on these guidelines, youth aged 14–17 are recommended to engage in 60 min/day moderate-to-vigorous PA (MVPA; with vigorous PA and muscle and bone strengthening activities incorporated at least 3 days/week), accumulate no more than 2 h/day of recreational ST, and get an average of 8–10 h/night of uninterrupted sleep [21]. Alarmingly, only 6% of Canadian youth aged 12–17 are estimated to meet all three guidelines [49]. About two-thirds fail to meet MVPA guidelines, three-quarters exceed 2 h/day of ST, and at least one-third sleep less than 8 h/day [49, 50].

Recent studies have examined the physical health benefits of meeting combinations of the 24-h Movement Guidelines for Children and Youth [51, 52]; however, their associations with mental health and illness remain relatively unexplored. In the Canadian 2013/2014 Health Behaviour in School-aged Children study, participants meeting any given guideline had lower “emotional problems” scores than their peers not meeting that recommendation [52]. Results were comparable regardless of which guideline or intermediate combination was met, with a dose-response pattern for the number of recommendations met. In a similar US representative study, youth meeting all three guidelines had lower depressive symptoms, but a dose-response pattern was not supported [53]. Also, higher depressive symptoms were associated with nonadherence to the sleep and MVPA guidelines, but not with weekday ST recommendations. Results of an Australian cross-sectional study point to differences by sex, with lower depressive symptoms associated with ST guideline adherence in females only, PA guideline adherence in males only, and adherence to the sleep guidelines among all youth [43]. Lastly, in a cohort study in Nova Scotia, Canada, meeting more lifestyle recommendations at age 10/11 predicted fewer mental illness-related physician visits over 8 years, with independent effects for ST and PA, but not for sleep guideline adherence [54]. Hence, while research tends to support mental health benefits of meeting more guidelines, evidence of the independent effects of MVPA, sleep, and ST guideline adherence is inconsistent.

Promoting adherence to the movement behaviours may offer a protective effect for mental health over adolescence; however, to best of our knowledge, only cross-sectional studies have been conducted on the combined 24-h movement behaviour guidelines and depression indicators, to date. Depressive symptoms can also lead to disturbed sleep, increased ST, and disengagement in PA [29–32, 55]. Moreover, to determine their independent effects, it is necessary examine the movement behaviours simultaneously given potential confounding. The objective of this study was to examine if changes in adherence to the Canadian 24-h Movement Behaviour Guidelines were associated with depressive symptoms over one year among youth.

## Methods

### Sample and design

This study used linked student data from Year 5 (Y_5_[2016–2017]) and 6 (Y_6_[2017–2018]) of the COMPASS Study. COMPASS (2012–2021) collects hierarchical longitudinal data from students in grades 9–12 and the Canadian secondary schools they attend [56]. All students attending participating schools were invited to participate using active-information passive-consent parental permission protocols.

During COMPASS Y_5_, new mental health measures were added to the COMPASS Student Questionnaire and piloted in 14 schools (5 British Columbia [BC], 9 Ontario) [57, 58]. The mental health measures were subsequently included in all COMPASS Student Questionnaires completed across the 124 schools participating in Y_6_ of COMPASS. Of the 14 schools in the Y_5_ pilot of the mental health measures, 12 schools also participated in Y_6_ (*N* = 3173), and 2356 (74.3%) students had depression scores at both Y_5_ and Y_6_. One student was removed for not indicating sex, and 63 students were excluded for missing movement data (57 PA, 11 ST, 11 sleep). The resultant sample comprised 2292 participants (72.2% of total linked) (see Table 1 for sample descriptives). In each school, the COMPASS Student Questionnaire was used to collect whole-school samples during class time. The cover page contains measures to create a unique code for each respondent to ensure anonymity, while allowing student data to be linked over multiple years. Further details of recruitment and retention (~ 80%), linkage, the overall COMPASS methods (www.compass.uwaterloo.ca), and the mental health pilot study are available elsewhere [56–60].

**Table 1:** Descriptive statistics in the 2-year linked COMPASS Mental Health Pilot Sample (Year 5: 2016/2017; Year 6: 2017/2018)

		Females	Males	Chi-square ***p***-value
		***N*** = 1229	***N*** = 1063	
		**% (N)**	**% (N)**	
Grade^a^	10	39.4 (484)	38.5 (409)	0.5238
	11	36.6 (450)	35.5 (377)	
	12	24.0 (295)	26.1 (277)	
Ethnicity	White	73.2 (900)	72.5 (771)	0.0684
	Black	2.0 (25)	2.0 (21)	
	Asian	8.2 (101)	8.7 (93)	
	Hispanic/Latin American	1.6 (20)	3.5 (37)	
	Other/Mixed	13.3 (164)	11.4 (121)	
BMI Classification	Underweight	1.1 (14)	1.6 (17)	0.0063
	“Normal-weight”	59.8 (735)	56.0 (595)	
	Overweight	12.3 (151)	15.2 (162)	
	Obesity	5.5 (68)	8.4 (89)	
	Missing BMI	21.2 (261)	18.8 (200)	
School-area household median income	$50,001-75,000	38.6 (475)	35.1 (373)	0.0951
	$75,001-100,000	55.3 (680)	57.2 (608)	
	>$100,000	6.0 (74)	7.7 (82)	
Urbanicity	Large Urban	53.2 (654)	54.8 (583)	0.2601
	Medium Urban	16.2 (199)	13.7 (146)	
	Small/Rural	30.6 (376)	31.4 (334)	
Transition in Movement Guideline Adherence (Y_5_–Y_6_)^b^				
MVPA	0–0	39.8 (489)	26.3 (280)	<.0001
	0–1	15.1 (186)	13.3 (141)	
	1–0	21.2 (261)	22.8 (242)	
	1–1	23.8 (293)	37.6 (400)	
ST	0–0	90.5 (1112)	94.3 (1002)	0.0005
	0–1	3.7 (46)	1.6 (17)	
	1–0	3.7 (45)	3.4 (36)	
	1–1	2.1 (26)	0.8 (8)	
Sleep Duration	0–0	50.2 (617)	48.0 (510)	0.4778
	0–1	11.6 (142)	11.0 (117)	
	1–0	17.3 (213)	17.5 (186)	
	1–1	20.9 (257)	23.5 (250)	
		**Mean (SD)**	**Mean (SD)**	**T-test p-value**
Transition in Total Guideline Adherence (Y_6_–Y_5_)		0.77 (0.72)	0.88 (0.83)	0.0006
Depressive symptoms		10.53 (6.57)	8.03 (5.55)	<.0001

Note: MVPA: moderate-to-vigorous physical activity. ST: screen time

^a^ Covariates from Year 6 (Y_6_:2017/2018).

^b^ Based on the Canadian 24-h Movement Guidelines (Tremblay et al., 2016)

### Measures

#### Depression symptoms

The 10-item *Center for Epidemiologic Studies Depression scale Revised (CESD-R-10)* [61–63] was included among the COMPASS mental health measures to assess depressive symptoms. Items assess characteristics of clinical depression, including negative affect, anhedonia, and somatic symptoms, such as “I felt everything I did was an effort”, “I could not get ‘going’”, difficulty concentrating, and feelings of hopelessness. Students were asked how often they experienced each symptom within the last 7 days, with the response options: “None or less than 1 day”, “1–2 days”, “3–4 days”, or “5–7 days”. Responses were scored from 0 to 3, respectively, and summed. Higher total scores indicate greater depressive symptoms. Psychometric properties have been evaluated in adolescent and adult populations [61, 64, 65]. Internal consistency was good (α = 0.78 Y_5_, 0.77 Y_6_).

#### Movement guideline adherence

*Sleep duration* was assessed by asking students how much time they usually spend sleeping per day. Responses were classified according to adherence to the guidelines of 8–10 h/day [21].

*ST* was assessed by asking the amount of time per day they usually spend engaging in different forms of screen use (watching/streaming TV shows/movies, playing video/computer games, talking on the phone, surfing the internet, texting/messaging/emailing). The sum of all forms of ST was dichotomized based on adherence to the guideline of no more than two hours/day of total recreational ST [21].

To determine *MVPA,* two items were used to assess how many minutes of moderate (i.e., lower intensity activities such as walking, biking to school, and recreational swimming) and hard (i.e., jogging, team sports, fast dancing, jump-rope, and any other physical activities that increase your heart rate and make you breathe hard and sweat) PA they accumulated on each of the last 7 days to calculate a daily *average*. Responses were classified according to whether they met the MVPA guideline of at least 60 min/day [21]. The PA and ST measures have been previously validated [66, 67].

*Transition in total guideline adherence* was determined by subtracting the number of guidelines that students adhered to in Y_5_ from their total guideline adherence in Y_6_. Possible scores ranged from − 3 to 3, with a positive score indicating that a student transitioned to meeting more Movement Guidelines (MVPA, ST, and/or sleep) in Y_6_ than in Y_5_, and a negative score means a student met fewer guidelines in Y_6_ than the previous year. Scores were treated as continuous variables.

#### Covariates

Student-level covariates included grade, ethnicity, and weight status (age- and sex-adjusted Body Mass Index [BMI; kg/m^2^] WHO classifications using student-reported height and weight). Missing BMI was included as a category, given the quantity of unreported weight data. School postal codes were cross-referenced with Statistics Canada data to determine school-area median average household income and urbanicity.

### Statistical analysis

Two conditional change models were conducted [68]. The first model tested change in adherence to the MVPA, ST, and sleep guidelines as predictors of Y_6_ depression scores, adjusting for Y_5_ depression and Y_6_ covariates. The second model tested transitions in total guideline adherence as predicting Y_6_ depressive symptoms at, adjusting for Y_5_ depression and Y_6_ covariates. Conditional change models are often used for two-wave panel data to take account of ‘regression towards the mean’ effects. Models were stratified by sex. Mixed models were used to account for school clustering by adding random intercepts at the school level. Analyses were conducted using SAS 9.4 (SAS Institute, Cary, NC, USA).

## Results

### Sample Descriptives

See Table 1 for descriptive statistics. The majority of the sample identified as white (73.2% females; 72.5% males) and over half had BMIs in the ‘normal-weight’ range. The sample primarily attended schools in areas with median household incomes of $50,000-100,000. Females had higher depressive scores than males (*p* < .0001).

### One-year change in guideline adherence

Males were more likely to meet the MVPA guidelines in both years than females. About 40% of females did not meet MVPA guidelines in either year, in comparison to 26.3% of males; whereas 37.6% and 23.8% of males and females met MVPA guidelines in both years, respectively. Consistent with known age and grade trends [69], more students transitioned from being active to not meeting the MVPA guidelines over one year than the reverse.

Most students did not meet the guidelines for total ST in either year (90.5% females; 94.3% males). Only 2.1% of females and 0.8% of males met the guidelines in both years.

About half of students reported sleep durations of less than 8 h in both, and approximately one-fifth reported sufficient sleep in both years. In line with known age trends over adolescence [50, 70], more students transitioned from adequate to short sleep than the reverse.

The mean difference in total of guideline adherence (Y_6_–Y_5_) was higher among males (*p* = .0006), indicating more males transitioned to meeting additional guidelines at follow-up than females.

### Conditional change models

See Table 2 for results modeling depressive symptoms by one-year change in adherence to the individual components (MVPA, ST, sleep) of the 24 h-movement guideline stratified by sex. Depression symptoms did not differ by changes in MVPA guideline adherence among females or males when controlling for prior year depression scores, covariates, and adherence transition to the other Movement Guidelines. For ST, females who transitioned from nonadherence to adherence reported lower depressive symptoms than students who continued to exceed guidelines. Results for continued ST guideline adherence in males and females, and for males transitioning from nonadherence to adherence for ST, were not interpreted due to the low frequency of students in these categories.

**Table 2:** Conditional change model of depression symptoms by one-year change in movement guideline adherence among students the 2-year linked COMPASS Mental Health Pilot Sample

	Females (N = 1229)				Males (N = 1063)			
	Est.	SE	95% CI	p-value	Est.	SE	95% CI	p-value
Grade (ref: 10)								
11	0.27	0.33	−0.38, 0.92	.4148	−0.39	0.33	− 1.04, 0.27	.2442
12	**−1.11**	**0.38**	**− 1.85, − 0.38**	**.0031**	**− 0.75**	**0.37**	**− 1.47, − 0.03**	**.0427**
Ethnicity (ref: White)								
Black	0.35	1.02	−1.64, 2.35	.7281	0.23	1.03	− 1.79, 2.25	.8263
Asian	−0.06	0.63	−1.29, 1.17	.9290	−0.06	0.56	−1.17, 1.05	.9162
Latin American/Hispanic	−0.08	1.14	−2.31, 2.15	.9451	0.66	0.79	−0.90, 2.22	.4059
Other/Mixed	0.16	0.43	−0.67, 1.00	.7017	0.84	0.46	−0.06, 1.73	.0671
BMI Classification (ref: “Normal-weight”)								
Underweight	0.42	1.35	−2.23, 3.07	.7583	0.02	1.16	−2.25, 2.28	.9884
Overweight	0.50	0.45	−0.38, 1.38	.2637	−0.39	0.42	−1.21, 0.43	.3472
Obesity	0.41	0.64	−0.84, 1.66	.5217	−0.71	0.53	−1.75, 0.34	.1854
Missing	0.68	0.36	−0.03, 1.39	.0622	0.54	0.39	−0.24, 1.31	.1737
Median household income (ref: $50,001-75,000)								
$75,001-100,000	−0.04	0.67	−1.35, 1.27	.9516	0.94	0.49	−0.01, 1.90	.0535
> $100,000	−1.34	0.92	−3.14, 0.46	.1454	0.50	0.62	−0.72, 1.72	.4243
Urbanicity (ref: Small/Rural)								
Medium Urban	−0.73	0.70	−2.11, 0.64	.2966	0.61	0.52	−0.40, 1.62	.2351
Large Urban	−0.44	0.66	−1.73, 0.86	.5097	−0.02	0.47	−0.93, 0.89	.9662
MVPA^a^								
0–1 vs. 0–0	−0.63	0.43	−1.47, 0.22	.1491	0.33	0.48	−0.61, 1.28	.4921
1–0 vs. 0–0	0.18	0.39	−0.58, 0.94	.6453	−0.17	0.41	−0.97, 0.63	.6779
1–1 vs. 0–0	−0.30	0.37	−1.03, 0.44	.4279	−0.54	0.37	−1.27, 0.20	.1516
1–1 vs. 1–0	−0.47	0.43	1.31, 0.36	.2679	−0.37	0.39	−1.12, 0.39	.3455
ST^ab^								
**0–1 vs. 0–0**	**−1.63**	**0.76**	**−3.11, −0.15**	**.0315**	**−2.75**	**1.14**	**−4.99, −0.51**	**.0161**
1–0 vs. 0–0	0.81	0.77	−0.70, 2.31	.2944	−0.36	0.79	−1.91, 1.19	.6511
1–1 vs. 0–0	−1.44	1.01	−3.41, 0.54	.1541	−2.63	1.66	−5.89, 0.63	.1143
1–1 vs. 1–0	−2.24	1.24	−4.67, 0.18	.0701	−2.27	1.83	−5.86, 1.32	.2157
Sleep Duration^a^								
**0–1 vs. 0–0**	**−1.76**	**0.47**	**−2.68, −0.85**	**.0002**	**−1.11**	**0.48**	**−2.04, − 0.17**	**.0210**
1–0 vs. 0–0	0.34	0.40	−0.45, 1.14	.3981	0.05	0.40	−0.74, 0.84	.8958
**1–1 vs. 0–0**	**−1.41**	**0.38**	**−2.15, −0.66**	**.0002**	**−1.62**	**0.37**	**−2.35, −0.90**	**<.0001**
**1–1 vs. 1–0**	**−1.75**	**0.46**	**−2.66, −0.84**	**.0002**	**−1.68**	**0.45**	**−2.57, −0.78**	**.0002**

Note: Model adjusted for prior year depressive symptoms and school clustering. MVPA: moderate-to-vigorous physical activity. ST: screen time. SE: Standard Error

^a^ Baseed on the Canadian 24-h Movement Guidelines (Tremblay et al., 2016)

^b^ Interpret ST results with caution given low frequency of students in 1–1 category (2.1% females; 0.8% males) and males in the 0–1 category (1.6%)

In both females and males, consistently meeting sleep duration guidelines was associated with lower depressive symptoms relative to individuals who consistently reported short sleep or those who transitioned from adequate to inadequate sleep. Also, females and males transitioning from inadequate to sufficient sleep reported lower depressive symptoms relative to their counterparts who consistently reported short sleep.

Table 3 presents results modeling depression scores as function of the difference in the total number of guidelines adhered to from the year prior. In females, meeting one or more guideline than the prior year was associated with lower depressive symptoms; however, transitions in total guideline adherence were not associated with depressive symptoms among males, when controlling for Y_5_ depression and Y_6_ covariates.

**Table 3:** Modeling depression symptoms (CESD-10-R) by one-year transition in total movement guideline adherence (MVPA, ST, sleep) among secondary school students the 2-year linked COMPASS Mental Health Pilot Sample

	Females (N = 1229)				Males (N = 1063)			
	Est.	SE	95% CI	p-value	Est.	SE	95% CI	p-value
Grade (ref: 10)								
11	0.37	0.33	−0.28, 1.02	.2642	−0.29	0.34	−0.95, 0.37	.3853
12	**−0.99**	**0.38**	**−1.72, −0.25**	**.0085**	−0.61	0.37	−1.33, 0.12	.1010
Ethnicity (ref: White)								
Black	0.21	1.02	−1.80, 2.21	.8408	0.32	1.04	−1.73, 2.36	.7626
Asian	0.19	0.62	−1.03, 1.41	.7583	0.12	0.57	−1.00, 1.23	.8352
Latin American/Hispanic	0.13	1.14	−2.10, 2.37	.9060	0.84	0.80	−0.73, 2.42	.2936
Other/Mixed	0.24	0.43	−0.60, 1.08	.5737	0.84	0.46	−0.07, 1.74	.0696
BMI Classification (ref: “Normal-weight”)								
Underweight	0.45	1.36	−2.21, 3.11	.7392	0.15	1.16	−2.13, 2.43	.8996
Overweight	0.54	0.45	−0.34, 1.42	.2328	−0.23	0.42	−1.06, 0.59	.5798
Obesity	0.53	0.64	−0.73, 1.79	.4115	−0.48	0.53	−1.52, 0.57	.3719
Missing	0.65	0.37	−0.06, 1.37	.0736	0.73	0.39	−0.05, 1.50	.0658
Median household income (ref: $50,001-75,000)								
$75,001-100,000	−0.05	0.64	−1.30, 1.21	.9526	0.91	0.49	−0.06, 1.88	.0652
> $100,000	−1.41	0.88	−3.13, 0.31	.1077	0.42	0.63	−0.81, 1.65	.5019
Urbanicity (ref: Small/Rural)								
Medium Urban	−0.81	0.67	−2.11, 0.50	.2251	0.59	0.52	−0.43, 1.61	.2593
Large Urban	−0.52	0.63	−1.75, 0.72	.4110	−0.07	0.47	−0.85, 0.99	.8826
Transition in total guideline adherence (Y_6_–Y_5_)^a^	**−0.71**	**0.17**	**−1.05, −0.37**	**<.0001**	−0.22	0.18	−0.57, 0.13	.2205

Note: Model adjusted for prior year depressive symptoms and school clustering. MVPA: moderate-to-vigorous physical activity. ST: screen time. SE: Standard Error.

^a^ Difference in total adherence to the Canadian 24-h Movement Guidelines (MVPA, total ST, sleep) (Tremblay et al., 2016) from year prior (Y6 total adherence minus Y5 total adherence)

## Discussion

The *Canadian 24-h Movement Behaviour Guidelines* [21] were developed based on systematic reviews demonstrating the physical, mental, and social benefits of sufficient MVPA [71], adequate sleep [72], and limited ST [73]; however, since their release, limited research has examined the combined and independent associations with mental health and illness symptoms, particularly over time. In a large sample of secondary school students from Ontario and BC, the current study examined one-year change in adherence to the 24-Hour Movement Behaviour Guidelines and depressive symptoms, adjusting for sociodemographic covariates and prior year depression scores. Adherence to the sleep guidelines emerged as the most consistent predictor of depression symptoms. Youth who consistently met sleep recommendations over one year reported lower depressive symptoms in comparison to youth reporting short sleep across both years and those who transitioned from guideline adherence to nonadherence. This study also provides prospective evidence of a link between ST and depressive symptoms; where females who transitioned to meet ST guidelines had lower scores than those who continued to exceed 2 h/day of total ST. However, no significant effect was found for one-year change in MVPA guideline adherence, when adjusting for the other guidelines, covariates, and prior year depression scores.

In the total adherence models, meeting more guidelines than the year prior predicted lower depressive symptoms in females only. Research has generally supported a cumulative effect, where the more guidelines adhered to, the larger the mental health benefit [52–54]. When sex differences have been found in previous studies, significant effects have tended to occur among females only [46, 47]. Results are unsurprising given known variations in the manifestation of depressive symptoms and movement behaviour engagement. That is, females report higher levels of internalizing symptoms and depressive disorders [2–4] and are generally less likely to meet sleep and MVPA guidelines than males [50, 74, 75]. Overall, effect sizes for depressive symptoms were modest, similar to previous studies of healthy youth populations [52]. However, with few youth consistently meeting recommendations, promoting guideline adherence has potential to make a substantial population-level impact on the prevention and early management of depressive symptoms [52, 76].

The independent associations for sleep, MVPA, and ST guideline adherence and depression vary across study. Similar to our results, an Australian cross-sectional study found sleep to be the only movement behaviour associated with depressive symptomatology in all youth, while inverse associations were found with ST guideline adherence in females and PA adherence in males [43]. In other cross-sectional research, Janssen et al. found adherence to each guideline had an inverse association with ‘emotional problem’ scores to an equivalent degree [52]; whereas, Zhu et al. found youth meeting recommendations for MVPA and sleep were less likely to have received a depression diagnosis, but no evidence of an association with ST [53]. Conversely, in a cohort study of children and youth, meeting ST and PA recommendations at age 10/11 predicted fewer mental illness-related physician visits over 8 years but meeting the sleep recommendations had no effect [54]. Methodology differences may contribute to inconsistent results, including cross-sectional versus prospective designs, types of ST included, and mental health indicators. Hayward et al. used self-reported depressive symptoms, while Janssen et al. employed a composite score (including depressive and other symptoms), Zhu et al. assessed parental reports of physician-diagnosed depression, and Loewen et al. linked survey data to administrative healthcare records [43, 53, 54]. Further prospective research of large youth populations remains necessary to determine the independent associations of adherence to each movement behaviour recommendation for various mental health and illness outcomes; however, overall, evidence suggests benefits for youth meeting more recommendations.

Results bolster calls to give sleep the same attention traditionally devoted to PA and screen use. Our findings augment evidence of an inverse association between sleep and depressive symptoms [39, 41–43, 45, 52, 53] and conflict with studies that found no prospective effect [54, 77] or an effect in females only [46, 47]. Once considered a core symptom or comorbidity of depressive disorders, some now identify sleep problems as both a prodromal manifestation and an independent risk factor for subsequent episodes, predicting the occurrence and outcome of depressive disorders [78]. In fact, given the inadequacy of treatments for youth depression [79], sleep therapy has been suggested as an intervention for adolescent MDD [80]. In addition to symptom management, further consideration from a population prevention standpoint is warranted, particularly given the proportion of youth sleeping less than 8 h/night.

Developmental changes in sleep have been suggested to partly account for the emergence of depressive symptoms and MDD over adolescence [39]. A natural shift towards later sleep onset contributes to a steady decline in youth sleep durations, coinciding with growing school pressures, increased extra-curricular activities, and less parental monitoring. As a result, youth bedtimes become progressively later, yet early school start times prohibit compensating with delayed wake times [81–84]. Aligning school schedules to adolescent sleep patterns appears an effective public health strategy for promoting longer sleep. Evidence suggests even modest school start time delays predict increased sleep durations [74, 85] as well as improved mental health and fewer emotional problems [85]. In addition to developmental trajectories, evidence suggests a population shift towards shorter average sleep durations occurred over the past several years [75, 86, 87], parallel with increased reports of tiredness and difficulties sleeping [88, 89] These trends coincide with increased reports of psychological distress and internalizing symptoms among youth [90] and further empathize the need to consider sleep in efforts to address youth mental health.

ST is the most debated of the movement behaviours, in terms of prospective links with mental health and illness. Some researchers have dismissed the effects as miniscule [28], while others argue a small risk of depression may result in substantial burden at the population level, considering the majority of youth exceed recommendations [76]. In the current study, so few youth reported ST guideline adherence that we are limited in interpreting results. Transitioning to meeting ST recommendations may have a modest effect on depressive symptoms in females. Several reviews have concluded research supports a positive relationship between leisure-time screen use and depressive symptoms, or internalizing symptoms more broadly, among adolescents [33, 34, 91–93]; however, much of this research has been cross-sectional. Proposed mechanisms for depression risk primarily focus on ST displacing more active, productive, or social activities. In this study, an association remained after adjustment for PA and sleep, suggesting depressive effects independent of their potential displacement; however, again, we are limited in interpretation. Other hypothesized mechanisms point to the context and content of ST engagement [76]. Sedentary behaviours often take place in solitude, potentially giving rise to rumination and feelings of isolation [76]; whereas PA typically occurs in the presence of peers among youth. The content itself could have a more direct effect, through social comparison or exposure to cyberbullying, for example. Further prospective research is needed exploring ways in which screens are used. Mixed findings in the literature may partially reflect inconsistencies in the form of ST assessed and included in determining guideline adherence, with prospective evidence varying by texting, computer, video game, or television time [24, 27, 76, 94].

Our MVPA results coincide with reviews of observational studies among largely healthy populations, in which prospective associations between PA and depression are typically small or null, as opposed to intervention studies among samples with clinically diagnosed depression [19, 20, 71, 95]. Only PA volume was examined, but the context likely has unique relationships with mental health [96–98]. Some evidence from young adults indicates MVPA is no longer associated with depressive symptoms when extracurricular activities such as team sports participation are accounted for [99], suggesting the mental health benefits relate more to positive social interaction and identity development. Many youth also report negative PA experiences, contributing to differences in PA and sports engagement by gender, body size, and age over adolescence [100–103]. Motives may also play a role. Appearance or weight loss PA motivations have been associated with negative psychosocial outcomes, as opposed to functional or enjoyment motivations [104]. Continued research is needed to explore various moderators in the relationship between PA and depression risk among youth.

Key strengths of this study include the linked data and adjustment for previous depression symptoms, as the reliance on cross-sectional evidence has been the most common criticism of previous research. The primary limitation pertains to self-report measures. As measures do not account for multi-tasking or for intermittent patterns of use, they likely overestimate total ST. The low frequency of students meeting ST guidelines may present power issues. Also, only two waves of linked data were available at time of this analysis. One-year change may not be optimal to understand effects over time. Lastly, COMPASS was not designed to be representative. The current sample was a pilot subsample of the larger study.

## Conclusion

To our knowledge, this study is the first to examine change in the adherence to the 24-h Movement Guidelines and depression symptoms over time in a population sample of youth. Adherence to the sleep recommendations emerged as the most consistent predictor of depression symptoms. Promoting adherence to the movement guidelines, particularly for sleep, should be considered a priority in the prevention and management of youth depressive symptoms. The decline in sleep duration over adolescence [50, 70] has long been recognized yet remains undervalued in efforts addressing youth mental health, despite simultaneous increases in depressive symptoms and MDE onset [39]. Depression in adolescence is associated with problematic substance use, suicidality, poor physical health, reduced academic achievement, and elevated risk for future episodes or other mental disorders continuing into adulthood [4, 12]. Given the large burden of depression [13, 14] and low adherence to the Movement Guidelines, supporting youth in continuing to meet recommendations over adolescence appears a worthwhile public health investment, with clear benefits for multiple aspects of youth physical, mental, and social health [50, 51, 71].

## Data Availability

COMPASS study data is available upon request through completion and approval of an online form: https://uwaterloo.ca/compass-system/information-researchers/data-usage-applicationThe datasets used during the current study are available from the corresponding author on reasonable request.

## Abbreviations

BC: British Columbia
COMPASS: The Cannabis use, Obesity, Mental health, Physical activity, Alcohol use, Smoking, and Sedentary behaviour study
MDE: Major depressive episode
MDD: Major depressive disorder
MVPA: Moderate-to-vigorous physical activity
PA: Physical Activity
ST: Screen Time

## References

[1] Kessler RC, Angermeyer M, Anthony JC, De Graff R, Demyttenaere K, Gasquet I (2007). Lifetime prevalence and age-of-onset distributions of mental disorders in the World Health Organization’s world mental health survey initiative. World Psychiatry.

[2] Cheung AH, Dewa CS (2006). Canadian community health survey: major depressive disorder and suicidality in adolescents. Health Policy.

[3] Findlay L (2017). Depression and suicidal ideation among Canadians aged 15 to 24. Health Rep.

[4] Naicker K, Galambos N, Zeng Y, Senthilselvan A, Colman I (2013). Social, demographic, and health outcomes in the 10 years following adolescent depression. J Adolesc Health.

[5] Ruston JL, Forcier M, Schectman RM (2002). J Am Acad Child Adolesc Psychiatry.

[6] Saluja G, Iachan R, Scheidt PC, Overpeck MD, Sun W, Giedd JN (2004). Prevalence of and risk factors for depressive symptoms among young adolescents. Arch Pediatr Adolesc Med.

[7] Birmaher B, Ryan ND, Williamson DE, Brent DA, Kaufman J, Dahl RE (1996). Childhood and adolescent depression: a review of the past 10 years. Part I. J Am Acad Child Psychiatry.

[8] Lewinsohn PM, Clarke GN, Seeley JR, Rohde P (1994). Major depression in community adolescents: age at onset, episode duration, and time to recurrence. J Am Acad Child Adolesc Psychiatry.

[9] Maletic V, Robinson M, Oakes T, Iyengar S, Ball SG, Russell J (2007). Neurobiology of depression: an integrated view of key findings. Int J Clin Pract.

[10] Kovacs Maria (1984). Depressive Disorders in Childhood. Archives of General Psychiatry.

[11] Costello EJ, Mustillo S, Erkanli A, Keeler G, Angold A (2003). Prevalence and development of psychiatric disorders in childhood and adolescence. Arch Gen Psychiatry.

[12] Johnson D, Dupuis G, Piche J, Clayborne Z, Colman I (2018). Adult mental health outcomes of adolescent depression: a systematic review. Depress Anxiety.

[13] Whiteford HA, Degenhardt L, Rehm J, Baxter AJ, Ferrari AJ, Erskine HE (2013). Global burden of disease attributable to mental and substance use disorders: findings from the global burden of disease study 2010. Lancet..

[14] Stephens T, Joubert N (2001). The economic burden of mental health problems in Canada. Chronic Dis Can.

[15] Olin B, Jayewardene A, Bunker M, Moreno F (2012). Mortality and suicide risk in treatment-resistant depression: an observational study of the long-term impact of intervention. PLoS One.

[16] Ekkekakis P (2013). Routledge handbook of physical activity and mental health.

[17] Mammen G, Faulkner G (2013). Physical activity and the prevention of depression: a systematic review of prospective studies. Am J Prev Med.

[18] Morres ID, Hatzigeorgiadis A, Stathi A, Comoutos N, Arpin-Cribbie C, Krommidas C, Theodorakis Y (2019). Aerobic exercise for adult patients with major depressive disorder in mental health services: a systematic review and meta-analysis. Depress Anxiety.

[19] Dale LP, Vanderloo L, Moore S, Faulkner G (2019). Physical activity and depression, anxiety, and self-esteem in children and youth: an umbrella systematic review. Ment Health Phys Act.

[20] Biddle SJH, Ciaccioni S, Thomas G, Vergeer I (2019). Physical activity and mental health in children and adolescents: an updated review of reviews and an analysis of causality. Ment Health Phys Act.

[21] Tremblay MS, Carson V, Chaput J-P, Connor GS, Dinh T, Duggan M (2016). Canadian 24-hour movement guidelines for children and youth: an integration of PA, sedentary behaviour, and sleep. Appl Physiol Nutr Metab.

[22] Prince SA, Melvin A, Roberts KC, Butler GP, Thompson (2020). Sedentary behaviour surveillance in Canada: trends, challenges and lessons learned. Int J Behav Nutr Phys Act.

[23] Leatherdale ST, Ahmed R (2011). Screen-based sedentary behaviours among a nationally representative sample of youth: are Canadian kids couch potatoes?. Chronic Dis Inj Can.

[24] Grøntved A, Singhammer J, Froberg K, Møller NC, Pan A, Pfeiffer KA, Kristensen PL (2015). A prospective study of screen time in adolescence and depression symptoms in young adulthood. Prev Med.

[25] Maras D, Flament MF, Murray M, Buchholz A, Henderson KA, Obeid N (2015). Screen time is associated with depression and anxiety in Canadian youth. Prev Med.

[26] Kremer P, Elshaug C, Leslie E, Toumbourou JW, Patton GC, Williams J (2014). Physical activity, leisure-time screen use and depression among children and young adolescents. J Sci Med Sport.

[27] Houghton S, Lawrence D, Hunter SC, Rosenberg M, Zadow C, Wood L, Shilton T (2018). Reciprocal relationships between trajectories of depressive symptoms and screen media use during adolescence. J Youth Adolesc.

[28] Orben A, Przybylski AK. Screens, teens, and psychological well-being: evidence from three time-use-diary studies. Psychol Sci. 2019:1–15. 10.1177/0956797619830329 PMC6512056 30939250

[29] Hayley AC, Skogen JC, Sivertsen B, Wold B, Berk M, Pasco JA, Overland S (2015). Symptoms of depression and difficulty initiating sleep from early adolescence to early adulthood: a longitudinal study. Sleep..

[30] Heffer T, Good M, Daly O, MacDonell E, Willoughby T (2019). The longitudinal association between social-media use and depressive symptoms among adolescents and young adults: an empirical reply to Twenge et al. (2018). Clin Psychol Sci.

[31] Lovato N, Short MA, Micic G, Hiller RM, Gradisar M (2017). An investigation of the longitudinal relationship between sleep and depressed mood in developing teens. Nat Sci Sleep.

[32] Raudsepp L, Vink K (2019). Longitudinal associations between sedentary behavior and depressive symptoms in adolescent girls followed 6 years. J Phys Act Health.

[33] Stiglic N, Viner RM (2019). Effects of screentime on the health and well-being of children and adolescents: a systematic review of reviews. BMJ Open.

[34] Liu M, Wu L, Yao S (2015). Dose-response association of screen time-based sedentary behaviour in children and adolescents and depression: a meta-analysis of observational studies. Br J Sports Med.

[35] Saunders TJ, Vallance JK (2017). Screen time and health indicators among children and youth: current evidence, limitations and future directions. Appl Health Econ Health Policy.

[36] Sund AM, Larsson B, Wichstrøm L (2011). Role of physical and sedentary activities in the development of depressive symptoms in early adolescence. Soc Psychiatry Psychiatr Epidemiol.

[37] Chattu VK, Manzar MD, Kumary S, Burman D, Spence DW, Pandi-Perumal SR (2018). The global problem of insufficient sleep and its serious public health implications. Healthcare (Basel).

[38] Perry GS, Patil SP, Presley-Cantrell LR (2013). Raising awareness of sleep as a healthy behavior. Prev Chronic Dis.

[39] Raniti MB, Allen NB, Schwartz O, Waloszek JM, Byrne ML, Woods MJ (2017). Sleep duration and sleep quality: associations with depressive symptoms across adolescence. Behav Sleep Med.

[40] Roberts RE, Roberts CR, Chan W (2008). Persistence and change in symptoms of insomnia among adolescents. Sleep..

[41] Bauducco SV, Flink IK, Jansson-Frojmark M, Linton SJ (2016). Sleep duration and patterns in adolescents: correlates and the role of daily stressors. Sleep Health.

[42] Fernandez-Mendoza J, Calhoun SL, Vgontzas AN, Li Y, Gaines J, Liao D, Bixler EO (2016). Insomnia phenotypes based on objective sleep duration in adolescents: depression risk and differential behavioral profiles. Brain Sci.

[43] Hayward J, Jacka FN, Skouteris H, Millar L, Strugnell C, Swinburn BA, Allender S (2016). Lifestyle factors and adolescent depressive symptomatology: associations and effect sizes of diet, physical activity and sedentary behaviour. Aust N Z J Psychiatry.

[44] Pasch KE, Laska MH, Lytle LA, Moe SG (2010). Adolescent sleep, risk behaviors, and depressive symptoms: are they linked?. Am J Health Behav.

[45] Roberts RE, Duong HT (2014). The prospective association between sleep deprivation and depression among adolescents. Sleep..

[46] Conklin A, Yao CA, Richardson CG (2018). Chronic sleep deprivation and gender-specific risk of depression in adolescents: a prospective population-based study. BMC Public Health.

[47] Mathew GM, Hale L, Chang A-M (2019). Sex moderates relationships among school night sleep duration, social jetlag, and depressive symptoms in adolescents. J Biol Rhythm.

[48] Chaput JP, Carson V, Gray CE, Tremblay MS (2014). Importance of all movement behaviors in a 24-hour period for overall health. Int J Environ Res Public Health.

[49] Statistics Canada. Health Fact Sheets. Physical activity and ST among Canadian children and youth, 2016 and 2017. https://www150.statcan.gc.ca/n1/pub/82-625-x/2019001/article/00003-eng.htm. Released April 17, 2019.

[50] Chaput J, Janssen I (2016). Sleep duration estimates of Canadian children and adolescents. J Sleep Res.

[51] Carson V, Chaput J, Janssen I, Tremblay MS (2017). Health associations with meeting new 24-hour movement guidelines for Canadian children and youth. Prev Med.

[52] Janssen I, Roberts KC, Thompson W (2017). Is adherence to the Canadian 24-hour movement behaviour guidelines for children and youth associated with improved indicators of physical, mental, and social health?. Appl Physiol Nutr Metab.

[53] Zhu X, Haegele JA, Healy S (2019). Movement and mental health: behavioral correlates of anxiety and depression among children of 6-17 years old in the US. Ment Health Phys Act.

[54] Loewen Olivia K., Maximova Katerina, Ekwaru John P., Faught Erin L., Asbridge Mark, Ohinmaa Arto, Veugelers Paul J. (2019). Lifestyle Behavior and Mental Health in Early Adolescence. Pediatrics.

[55] Gunnell KE, Flament MF, Buchholz A, Henderson KA, Obeid N, Schubert N, Goldfield GS (2016). Examining the bidirectional relationship between physical activity, screen time, and symptoms of anxiety and depression over time during adolescence. Prev Med.

[56] Leatherdale ST, Brown KS, Carson V, Childs RA, Dubin JA, Elliott SJ (2014). The COMPASS study: a longitudinal hierarchical research platform for evaluating natural experiments related to changes in school-level programs, policies and built environment resources. BMC Public Health.

[57] Patte KA, Bredin C, Henderson J, Elton-Marshall T, Faulkner G, Sabiston C, et al. Development of a mental health module for the COMPASS system: improving youth mental health trajectories. Part 1: Draft development and design. Waterloo: University of Waterloo; 2017. Available at: www.compass.uwaterloo.ca.

[58] Patte KA, Bredin C, Henderson J, Elton-Marshall T, Faulkner G, Sabiston C, et al. Development of a mental health module for the COMPASS system: improving youth mental health trajectories. Part 2: Pilot test and focus group results. Waterloo: University of Waterloo; 2017. Available at: www.compass.uwaterloo.ca.

[59] Reel R, Bredin C, Battista K, Leatherdale ST. COMPASS year 5 and 6 school recruitment and retention. Tech Rep Ser. 2018;5(1) Waterloo, Ontario: University of Waterloo. Available at: https://uwaterloo.ca/compass-system/publications#technical.

[60] Qian W, Battista K, Bredin C, Brown KS, Leatherdale ST. Assessing longitudinal data linkage results in the COMPASS study. Tech Rep Ser. 2015;3(4) Waterloo, Ontario: University of Waterloo. Available at: https://uwaterloo.ca/compass-system/publications#technical.

[61] Andresen EM, Malmgren JA, Carter WB, Patrick DL (1994). Screening for depression in well older adults: evaluation of a short form of the CES-D. Am J Prev Med.

[62] Radloff LS (1977). The CES-D scale: a self-report depression scale for research in the general population. Appl Psychol Meas.

[63] Zhang W, O’Brien N, Forrest JI, Salters K, Patterson TL, Montaner JS (2012). Validating a shorted depression scale (10 item CES-D) among HIV-positive people in British Columbia, Canada. PLoS One.

[64] Bradley KL, Bagnell AL, Brannen CL (2010). Factorial validity of the center for epidemiological studies depression 10 in adolescents. Issues Ment Health Nurs.

[65] Haroz EE, Ybarra M, Eaton WW (2014). Psychometric evaluation of a self-report scale to measure adolescent depression: the CESDR-10 in two national adolescent samples in the United States. J Affect Disord.

[66] Wong S, Leatherdale ST, Manske S (2006). Reliability and validity of a school-based PA questionnaire. Med Sci Sports Exerc.

[67] Leatherdale ST, Laxer RE, Faulkner G. Reliability and validity of the PA and sedentary behaviour measures in the COMPASS study. COMPASS Tech Rep Ser. 2014;2(1) Waterloo, Ontario: University of Waterloo. Available at: www.compass.uwaterloo.ca.

[68] Aickin M (2016). Dealing with change: using the conditional change model for clinical research. Perm J.

[69] Dumith SC, Gigante DP, Domingues MR, Kohl HW (2011). Physical activity change during adolescence: a systematic review and a pooled analysis. Int J Epidemiol.

[70] Gradisar M, Gardner G, Dohnt H (2011). Recent worldwide sleep patterns and problems during adolescence: a review and meta-analysis of age, region, and sleep. Sleep Med.

[71] Poitras VJ, Gray CE, Borghese MM, Carson V, Chaput J-P, Janssen I (2016). Systematic review of the relationships between objectively measured physical activity and health indicators in school-aged children and youth. Appl Physiol Nutr Metab.

[72] Chaput J, Gray C, Poitras V, Carson V, Gruber R, Olds T (2016). Systematic review of the relationships between sleep duration and health indicators in school-aged children and youth. Appl Physiol Nutr Metab.

[73] Carson V, Hunter S, Kuzik N, Gray CE, Poitras VJ, Chaput J (2016). Systematic review of sedentary behaviour and health indicators in school-aged children and youth: an update. Appl Physiol Nutr Metab.

[74] Patte KA, Qian W, Cole A, Faulkner G, Chaput J, Carson V, Leatherdale ST (2018). School start time changes in the COMPASS study: associations with youth sleep duration, physical activity, and screen time. Sleep Med.

[75] Patte KA, Qian W, Leatherdale ST (2017). Sleep duration trends and trajectories among youth in the COMPASS study. Sleep Health.

[76] Khouja JN, Munafò MR, Tilling K, Wiles NJ, Joinson C, Etchells P (2019). Is screen time associated with anxiety or depression in young people? Results from a UK birth cohort. BMC Public Health.

[77] Mazzer K, Boersma K, Linton SJ (2019). A longitudinal view of rumination, poor sleep and psychological distress in adolescents. J Affect Disord.

[78] Fang H, Tu S, Sheng J, Shao A (2019). Depression in sleep disturbance: a review on a bidirectional relationship, mechanisms and treatment. J Cell Mol Med.

[79] Clarke G, Harvey AG (2012). The complex role of sleep in adolescent depression. Child Adolesc Psychiatr Clin N Am.

[80] Holtmann M, Mokras L, Kirschbaum-Lesch KM, Plener PL, Ruckes C (2018). Adolescent depression: study protocol for a randomized, controlled, double-blind multicenter parallel group trial of Bright Light Therapy in a naturalistic inpatient setting (DeLight). Trials.

[81] Bartel KA, Gradisar M, Williamson P (2015). Protective and risk factors for adolescent sleep: a meta-analytic review. Sleep Med Rev.

[82] Bartel K, Williamson P, van Maanen A, Cassoff J, Meijer AM, Oort F (2016). Protective and risk factors associated with adolescent sleep: findings from Australia, Canada, and The Netherlands. Sleep Med.

[83] Crowley SJ, Acebo C, Carskadon MA (2007). Sleep, circadian rhythms, and delayed phase in adolescence. Sleep Med.

[84] Maslowsky J, Ozer EJ (2014). Developmental trends in sleep duration in adolescence and young adulthood: evidence from a national United States sample. J Adolesc Health.

[85] Chan NY, Zhang J, Yu MW, Lam SP, Li SX, Kong AP (2017). Impact of a modest delay in school start time in Hong Kong school of adolescents. Sleep Med.

[86] Keyes K, Maslowsky J, Hamilton A, Schulenberg J (2015). The great sleep recession: changes in sleep duration among US adolescents, 1991-2012. Pediatrics..

[87] Matricciani L, Olds T, Petkov J (2012). In search of lost sleep: secular trends in the sleep time of school-aged children and adolescents. Sleep Med Rev.

[88] Chaput JP (2018). Prevalence of insomnia for Canadians aged 6 to 79. Stat Can.

[89] Kronholm E, Puusniekka R, Jokela J, Villberg J, Urrila AS, Paunio T (2015). Trends in self-reported sleep problems, tiredness and related school performance among Finnish adolescents from 1984 to 2011. J Sleep Res.

[90] Comeau J, Georgiades K, Duncan L, Wang L, Boyle M (2019). 2014 Ontario child health study team. Changes in the prevalence of child and youth mental disorders and perceived need for professional help between 1983 and 2014: evidence from the Ontario child health study. Can J Psychiatr.

[91] Costigan SA, Barnett L, Plotnikoff RC, Lubans DR (2013). The health indicators associated with screen-based sedentary behavior among adolescent girls: a systematic review. J Adolesc Health.

[92] Hoare E, Milton K, Foster C, Allender S (2016). The associations between sedentary behaviour and mental health among adolescents: a systematic review. Int J Behav Nutr Phys Act.

[93] Zhai L, Zhang Y, Zhang D (2015). Sedentary behaviour and the risk of depression: a meta-analysis. Br J Sports Med.

[94] Wu X, Kirk SFL, Ohinmaa A, Veugelers P (2018). Influence of physical activity, sedentary behavior, and diet quality in childhood on the incidence of internalizing and externalizing disorders during adolescence: a population-based cohort study. Ann Epidemiol.

[95] Korczak DJ, Madigan S, Colasanto M (2017). Children's physical activity and depression: a meta-analysis. Pediatrics..

[96] Dóre I, O’Loughlin JL, Beauchamp G, Martineau M, Fournier L (2016). Volume and social context of physical activity in association with mental health, anxiety and depression among youth. Prev Med.

[97] Evans MB, Allan V, Erickson K, Martin LJ, Budziszewski R, Cote J (2017). Are all sports activities equal? A systematic review of how youth psychosocial experiences vary across differing sport activities. Br J Sports Med.

[98] Lubans D, Richards J, Hillman C, Faulkner G, Beauchamp M, Nilsson M (2016). Physical activity for cognitive and mental health in youth: a systematic review of mechanisms. Pediatrics..

[99] Brunet J, Sabiston CM, O’Loughlin E, Chaiton M, Low NCP, O’Loughlin JL (2014). Symptoms of depression are longitudinally associated with sedentary behaviors among young men but not among young women. Prev Med.

[100] Tiggemann M, Slater A (2011). Gender differences in adolescent sport participation, teasing, self-objectification and body image concerns. J Adolesc.

[101] Deforche BI, De Bourdeaudhuij IM, Tanghe AP (2006). Attitude toward physical activity in normal-weight, overweight and obese adolescents. J Adolesc Health.

[102] Zabinski MF, Saelens BE, Stein RI, Hayden-Wade HA, Wilfley DE (2003). Overweight children's barriers to and support for physical activity. Obes Res.

[103] Crane J, Temple V (2014). A systematic review of dropout from organized sport among children and youth. Eur Phys Educ Rev.

[104] Strelan P, Mehaffey SJ, Tiggemann M (2003). Brief report: self-objectification and esteem in young women: the mediating role of reasons for exercise. Sex Roles.

[105] Patte KA, Faulkner G, Duncan M, Qian W, Leatherdale ST. Are changes in adherence to the 24-hour movement guidelines associated with depression and anxiety symptoms among youth? Oral presentation at the *International Society of Behavioral Nutrition and Physical Activity (ISBNPA)* Annual Conference, Prague, Czech Republic, June 6, 2019.

